# Individual- and Area-Level Incarceration and Mortality

**DOI:** 10.1001/jamanetworkopen.2025.13537

**Published:** 2025-06-03

**Authors:** Utsha G. Khatri, Jahn K. Hakes, David Buckler, Alexis Zebrowski, Tyler Winkelman

**Affiliations:** 1 Department of Emergency Medicine, Icahn School of Medicine at Mount Sinai, New York, New York; 2 US Census Bureau, Suitland, Maryland; 3 Division of General Internal Medicine, Hennepin Healthcare, Minneapolis, Minnesota; 4 Health, Homelessness, and Criminal Justice Lab, Hennepin Healthcare Research Institute, Minneapolis, Minnesota

## Abstract

**Importance:**

The US has the highest incarceration rates in the developed world. The harms of incarceration have long-term health implications, including increased mortality. Existing studies of incarceration-related mortality are limited by data sources and design.

**Objective:**

To examine the associations between both individual- and area-level incarceration rates with all-cause and overdose mortality in the US.

**Design, Setting, and Participants:**

This cohort study used data from the Mortality Disparities in American Communities (MDAC) study, linking over 3 million 2008 American Community Survey (ACS) respondents to National Death Index data from the respondents’ 2008 interview date through December 31, 2019, or their date of death, and county incarceration data from the Vera Institute of Justice. The sample included US adults 18 years or older, representing individuals in group quarters such as prisons and jails but excluding those in counties lacking jail incarceration rate data. Data were analyzed from July 5, 2023, to November 10, 2024.

**Exposure:**

Individual incarceration status at the time of the ACS survey and county jail incarceration rates.

**Main Outcomes and Measures:**

The outcomes of interest were all-cause mortality and overdose mortality, assessed through time-to-event analyses. Cox proportional hazard models were used to estimate mortality risks, adjusting for individual- and county-level characteristics. ACS survey weights were applied so that the final sample represents the US adult population.

**Results:**

The study includes a total of 3 255 000 individuals (51.3% female), of whom 45 000 (0.93%) were incarcerated at the time of the 2008 ACS administration. The mean (SD) county jail incarceration rate was 372 (358) per 100 000 people. During the study period, 431 000 individuals (11.6%) died from any cause, and 5500 (0.2%) died from overdoses. Incarcerated individuals had a higher risk of all-cause mortality (hazard rate [HR], 1.39 [95% CI, 1.33-1.45]) and an increased risk of overdose mortality (HR, 3.08 [95% CI, 2.70-3.52]) compared with nonincarcerated individuals. A 10% increase in county jail incarceration rates was associated with 4.6 (95% CI, 3.8-5.5) additional all-cause deaths per 100 000 people.

**Conclusions and Relevance:**

In this cohort study of 3.26 million individuals in the US, results highlighted the dual burden of incarceration on health outcomes. Individuals who were incarcerated faced significantly higher risks of death, particularly from overdoses, and elevated county incarceration rates exacerbated individual-level mortality risks. These findings suggest the need for reforms in criminal justice and public health policies to address these elevated risks and their widespread implications.

## Key Points

**Question:**

Are individual incarceration status and county incarceration rates associated with all-cause and overdose mortality in the US?

**Findings:**

In a nationally representative cohort study of approximately 3.26 million adults observed from 2008 through 2019, individuals incarcerated at the time of the survey experienced a 39% higher risk of all-cause mortality and more than 3 times the risk of overdose mortality compared with nonincarcerated individuals. County incarceration rates were also associated with increased all-cause mortality risks, even for nonincarcerated residents.

**Meaning:**

These findings suggest that incarceration significantly increases the risk of death for individuals and community populations, underscoring the need for reforms in criminal justice and public health policies to address the severe health risks associated with incarceration.

## Teaser

This cohort study examines the associations between both individual- and area-level incarceration rates with all-cause and overdose mortality in the US.

## Introduction

The US has the highest incarceration rate in the developed world, at 583 per 100 000 residents.^1^ More than 5 million US citizens are incarcerated or on probation or parole. This burden disproportionately affects American Indian or Alaska Native, Black, and other racially or ethnically minoritized groups and communities experiencing poverty.^2,3^ The harms of incarceration have long-term health implications.^4,5,6,7,8,9^ In single-state studies, the risk of death after release is significantly higher among recently imprisoned individuals compared with the general population. A Washington State study found the risk of death within 2 weeks of release was 12.7 (95% CI, 9.2-17.4) times that of other residents.^10,11^ National studies show that higher county jail incarceration rates are linked to increased mortality at the population level. One study estimated that for every 1 per 1000 increase in the local jail incarceration rate, death rates from infectious and chronic respiratory diseases rose by 6.5% and 5.0%, respectively.^12^

While a growing body of literature reveals insights into incarceration-related mortality, existing studies are limited by data sources and design. Several rely on synthetic controls in the general population rather than a true control group from the same dataset.^10,11^ Moreover, most do not control for both individual and area-level factors known to confound incarceration-mortality associations.^13^ Individual-level data are often regional and lack national representation, limiting generalizability. These gaps underscore the urgent need for a national study examining incarceration and mortality to guide policies on the overdose crisis and health care access during incarceration and reentry.

The objective of this study was to examine the associations of individual- and area-level incarceration rates with all-cause and overdose mortality in the US. We used county incarceration data linked to the Mortality Disparities in American Communities (MDAC) study, a nationally representative cohort of the 2008 American Community Survey (ACS) participants followed up for cause of death through 2019.^14^ We provide a comprehensive survival analysis incorporating both individual and area-level factors to examine incarceration’s association with mortality on a national scale. We hypothesized that both individual incarceration and county incarceration rates would be associated with increased all-cause and overdose mortality.

## Methods

The Mount Sinai Institutional Review Board determined this study to be exempt from human participants research regulations. The Office of Management and Budget approved collection and analysis of deidentified ACS data. To meet Title 13 privacy protection requirements, a research proposal was submitted to the MDAC steering committee, with assistance from a Census analyst, a process available to all investigators. The US Census Bureau has ensured appropriate access and use of confidential data and has reviewed these results for disclosure avoidance protection. We followed the Strengthening the Reporting of Observational Studies in Epidemiology (STROBE) guidelines to ensure transparent reporting of this cohort study.

### Data Sources

We used data from the MDAC project, which aims to facilitate research on mortality disparities by social and economic characteristics.^14^ The MDAC data linked the 2008 ACS to mortality data obtained using the National Death Index (NDI) and other sources from the respondents’ 2008 ACS interview date through December 31, 2019, or their death. The MDAC provides individual-level data on mortality, cause and date of death, interview date, and self-reported demographic and socioeconomic characteristics, including race and ethnicity. Adjusting for race and ethnicity helps account for structural and systemic inequities that independently influence both incarceration rates and mortality outcomes. Race and ethnicity were categorized as Hispanic, non-Hispanic American Indian or Alaska Native, non-Hispanic Asian, non-Hispanic Black, non-Hispanic White, and other (including those who self-identified as non-Hispanic and multiple races).

MDAC data permit survival analysis to determine the association with mortality risk from 1 variable (eg, incarceration on the date of the ACS interview) while controlling for others, such as age, sex, income, and educational attainment. The model can also adjust for effects from area-level poverty rate, population density, or incarceration rates. We used Cox proportional hazards regression to analyze survival, right-censoring surviving respondents at follow-up end on December 31, 2019. The period at risk was calculated from the interview date (2008) until either the date of death or the study’s end. Person-time was computed for all individuals, and follow-up duration was determined for each participant.

To control for county-level incarceration rates, we linked Vera Institute of Justice county-level data from 2008 to 2018 from the Bureau of Justice Statistics, Deaths in Custody Reporting Program, and Annual Survey of Jails, using linear interpolation for missing data. About 300 counties without recorded jail populations were excluded. County-level data on mean household size, poverty rates, and population density were taken from ACS data from 2011 to 2018. When specific year data were unavailable, the closest year’s data were used, with data preceding the event year used whenever possible.

### Study Sample

The MDAC study contains sociodemographic information for more than 4.51 million respondents to the 2008 ACS. Of these, 4.48 million could be matched to NDI death certificates or identified as alive (using first name, last name, social security number, and date of birth), with approximately 91% matched through social security numbers and the remaining linked using name and date of birth. Local variables on county jail incarceration rates came from the Vera Institute of Justice Incarceration Trends Dataset, described elsewhere.^15^ Information on the county-level percentage of the Black population was obtained from the ACS 5-Year Summary Files for 2008 to 2012 and 2015 to 2019.^16^ Vera Institute of Justice and ACS Summary File data were linked to MDAC through county Federal Information Processing Standard (FIPS) codes, with minor FIPS code changes corrected during follow-up.

Missing data were handled using complete case analysis, where observations with missing values for key variables were excluded. The initial mortality-linked MDAC dataset included 4.48 million participants across 3100 county equivalents in the 50 states and Washington, DC. We excluded individuals younger than 18 years (1.032 million [23.0%]), those with ACS records missing NDI linkage data (33 000 [0.7%]), and those with ACS records missing Vera Institute of Justice incarceration data (110 000 [2.5%]). The final linked dataset had 3.26 million individuals across 2900 county equivalents. Census Bureau statisticians adjusted ACS survey weights so the final sample represented the US adult population, including populations in Armed Services and group quarters.

### Exposure and Outcomes

We examined incarceration exposure at both the individual and population levels. Incarceration at the individual level was identified using the group quarters variables in the ACS. ACS defines group quarters as “places where people live or stay, in a group living arrangement, that is owned or managed by an entity or organization providing housing and/or services for the residents.” Group quarters are further defined as institutional group quarters, which include correctional facilities, juvenile facilities, skilled-nursing facilities, and psychiatric hospitals, and noninstitutional group quarters, which include student housing, military quarters, and shelters for the unhoused. For our analysis, incarceration at the time of the ACS survey was identified by any respondent coded as living in a correctional facility.

Population-level incarceration exposure was obtained through the Vera Institute of Justice Incarceration Trends Dataset. We modeled total jail population rate (TJPR) as a continuous variable in the natural logarithm form such that the residuals of the model would be normally distributed. We linked ACS respondents to county incarceration rates using FIPS code.

The main outcome was all-cause mortality. The secondary outcome was drug overdose mortality, identified using Centers for Disease Control and Prevention definitions and codes X40 to X44, X60 to X64, X85, and Y10 to Y14 from *International Statistical Classification of Diseases, Tenth Revision*.^17^

### Statistical Analysis

Data were analyzed from July 5, 2023, to November 10, 2024, using Stata statistical software (version 18; StataCorp). We compared the baseline characteristics of individuals incarcerated during the 2008 ACS survey with those not incarcerated using unpaired 2-sample *t* tests for proportions. The inequality of proportions within categorical groups was confirmed with χ^2^ tests and supported by *t* tests for specific categories. A significance threshold of 2-sided *P* < .05 was applied to all analyses, and 95% CIs were reported. Cox proportional hazards models examined the association between individual and population level incarceration and mortality. The foundation is a univariable model of survival controlling for TJPR. A second model adds individual characteristics, while a third adds both individual and county characteristics in multivariable adjusted hazard models. All covariates were retained based on clinical significance. Individual-level covariates included sex, age, race and ethnicity, marital status, educational attainment, employment, and household income (eTable 1 in Supplement 1). County-level covariates included racial composition (percentage Black), mean household size, population density, and poverty rate.

To test sensitivity, we also examined mortality effects of county incarceration rates coded by quartiles, with quartile 1 having the lowest rate and quartile 4 the highest. Additionally, we constructed Kaplan-Meier style survival curves comparing cumulative mortality during follow-up by (1) quartile of TJPR, (2) incarceration status at time of interview, (3) quartile of TJPR for those who were not incarcerated, and (4) quartile of TJPR for those who were incarcerated.

## Results

The study included a total of 3 255 000 individuals (51.3% female and 48.7% male; 13.8% Hispanic, 0.6% non-Hispanic American Indian or Alaska Native, 4.5% non-Hispanic Asian, 11.7% non-Hispanic Black, 68.4% non-Hispanic White, and 1.1% other), 45 000 (0.93%) of whom were incarcerated at the 2008 ACS. Compared with individuals who were not incarcerated, incarcerated individuals were more likely to be male (90.4% vs 48.3%), younger (≤35 years, 54.3% vs 31.7%), Hispanic (20.0% vs 13.7%), non-Hispanic American Indian or Alaskan Native (1.6% vs 0.6%), non-Hispanic Black (38.4% vs 11.4%), and never married (58.7% vs 26.3%) and to have less than a high school–level education (39.6% vs 15.1%) (*P* < .001 for all comparisons) (Table). A total of approximately 35.18 million person-years were analyzed, with a mean (SD) follow-up duration of 10.81 (2.21) years.

**Table.** Demographic Characteristics of Study Population^a^

Characteristic	Study population, No. (%) [SD of proportion]^b^			Comparison of incarcerated vs nonincarcerated groups	
	All (N = 3 255 000)	Incarcerated at time of survey (n = 45 000)	Not incarcerated at time of survey (n = 3 210 000)	Unpaired *t* test	*P* value
**Individual**					
Sex, No. (%) [proportion]					
Female	1 694 000 (51.3) [0.50]	4100 (9.6) [0.30]	1 690 000 (51.7) [0.50]	185.0	<.001
Male	1 561 000 (48.7) [0.50]	41 000 (90.4) [0.30]	1 520 000 (48.3) [0.50]	−185.0	
Age, y					
18-25	378 000 (14.2) [0.35]	10 000 (22.5) [0.42]	368 000 (14.1) [0.35]	−73.9	<.001
26-35	493 000 (17.7) [0.38]	14 500 (31.8) [0.47]	479 000 (17.6) [0.38]	−98.1	<.001
36-45	585 000 (19.2) [0.39]	11 500 (26.1) [0.44]	574 000 (19.1) [0.39]	−43.2	<.001
46-65	1 199 000 (33.6) [0.47]	8600 (18.7) [0.39]	1 191 000 (33.8) [0.47]	78.9	<.001
66-85	538 000 (13.6) [0.34]	400 (0.88) [0.09]	537 000 (13.7) [0.34]	90.0	<.001
≥86	61 000 (1.7) [0.13]	Suppressed	61 000 (1.7) [0.13]	NA	NA
Race and ethnicity					
Hispanic	336 000 (13.8) [0.35]	9000 (20.0) [0.40]	328 000 (13.7) [0.34]	−66.7	<.001
Non-Hispanic American Indian or Alaskan Native	23 000 (0.6) [0.08]	750 (1.6) [0.13]	22 000 (0.6) [0.08]	−25.5	<.001
Non-Hispanic Asian	128 000 (4.5) [0.21]	400 (0.8) [0.09]	127 000 (4.5) [0.21]	33.4	<.001
Non-Hispanic Black	294 000 (11.7) [0.32]	17 500 (38.4) [0.49]	276 000 (11.4) [0.32]	−220.0	<.001
Non-Hispanic White	2 439 000 (68.4) [0.47]	16 500 (36.8) [0.48]	2 422 000 (68.7) [0.46]	191.0	<.001
Other^c^	36 000 (1.1) [0.11]	1100 (2.3) [0.15]	35 000 (1.1) [0.11]	−26.3	<.001
Marital status					
Married	1 907 000 (53.5) [0.50]	7600 (17.2) [0.38]	1 899 000 (53.9) [0.50]	182.0	<.001
Widowed	225 000 (6.2) [0.24]	750 (1.6) [0.13]	224 000 (6.3) [0.24]	44.3	<.001
Divorced	351 000 (11.3) [0.32]	7800 (17.3) [0.38]	343 000 (11.3) [0.32]	−45.2	<.001
Separated	61 000 (2.3) [0.15]	2350 (5.3) [0.22]	58 500 (2.3) [0.15]	−52.9	<.001
Never married	711 000 (26.6) [0.44]	26 500 (58.7) [0.49]	685 000 (26.3) [0.44]	−190.0	<.001
Educational attainment					
Less than high school	455 000 (15.3) [0.36]	18 000 (39.6) [0.49]	437 000 (15.1) [0.36]	−160.0	<.001
High school diploma	954 000 (28.6) [0.45]	18 000 (40.3) [0.49]	936 000 (28.5) [0.45]	−51.7	<.001
Some college	980 000 (30.4) [0.46]	7800 (17.1) [0.38]	972 000 (30.5) [0.46]	60.0	<.001
Degree	866 000 (25.6) [0.44]	1400 (3.0) [0.17]	864 000 (25.8) [0.44]	115.0	<.001
Employment					
Unemployed	115 000 (4.1) [0.20]	NA	115 000 (4.1) [0.20]	NA	NA
Employed	2 021 000 (64.0) [0.48]	NA	2 021 000 (64.6) [0.48]	NA	NA
Not in labor force	1 074 000 (31.0) [0.46]	NA	1 074 000 (31.2) [0.46]	NA	NA
Household poverty^d^					
<100%	321 000 (11.5) [0.32]	NA	321 000 (11.6) [0.32]	NA	NA
100%-199%	546 000 (17.4) [0.38]	45 000 (100) [baseline]	501 000 (16.6) [0.37]	NA	NA
200%-399%	999 000 (30.6) [0.46]	NA	999 000 (30.9) [0.46]	NA	NA
400%-899%	1 084 000 (31.7) [0.47]	NA	1 084 000 (32.0) [0.47]	NA	NA
≥900%	304 000 (8.8) [0.28]	NA	304 000 (8.9) [0.28)	NA	NA
**County**					
Black population, mean (SD), %	12.7 (12.9)	14.2 (14.8)	12.7 (12.9)	−45.9	<.001
Total jail population rate per 100 000 in 2008-2018, mean (SD)	372 (358)	493 (915)	371 (349)	−63.3	<.001
Household size, mean (SD), No. of persons	2.62 (0.26)	2.62 (0.28)	2.62 (0.26)	−28.0	<.001
Population per square mile, mean (SD)	3240 (11 900)	1280 (6710)	3260 (11 900)	26.9	<.001
County poverty rate, mean (SD)^e^	14.8 (5.1)	16.5 (5.4)	14.8 (5.1)	−81.5	<.001
Incarcerated at interview, No. (%) [proportion]	45 000 (0.93) [0.10]	45 000 (100)	0	NA	NA
Deceased	431 000 (11.6) [0.32]	3500 (7.7) [0.27]	428 000 (11.6) [0.32]	34.7	<.001
Overdose death	5500 (0.2) [0.04]	550 (1.2) [0.11]	5000 (0.2) [0.04]	−55.4	<.001

Abbreviation: NA, not applicable.

^a^ Results approved by US Census Bureau Disclosure Review Board (approval number CBDRB-FY24-CES028-003).

^b^ All percentages are weighted so that the sample represents the US adult population.

^c^ Other included those respondents who self-identified as non-Hispanic and checked multiple race boxes, and thus could not be assigned uniquely to either/any of those races.

^d^ Based on poverty thresholds set annually by the US Census Bureau. The ACS assigns poverty status to each household based on the total income of the household relative to these thresholds (eg, <100% means household income is below the federal poverty threshold [considered in poverty]; ≥100% means household income is above the poverty threshold).

^e^ Indicates percentage of people in a county whose income falls below 100% of the federal poverty threshold.

**Figure 1.**
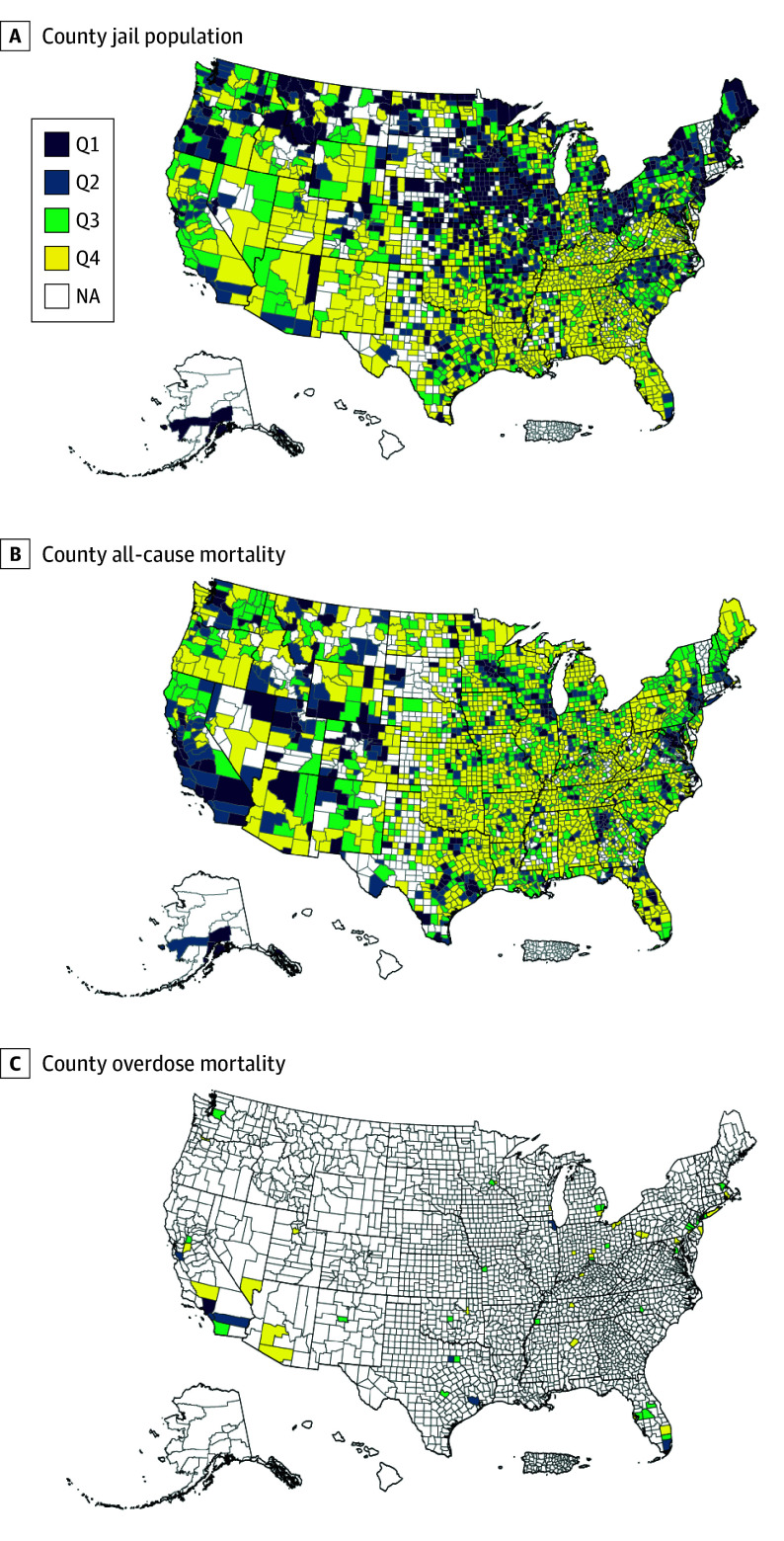
US Counties by Quartile of Total Jail Population Rate, All-Cause Mortality, and Overdose Mortality Results approved by US Census Bureau Disclosure Review Board (approval number CBDRB-FY24-CES028-003). Q indicates quartile; Q1 is the lowest and Q4 the highest.

At the county level, incarcerated individuals were more likely to be in counties with a higher percentage of Black residents (14.8% vs 12.9%), higher mean (SD) total jail population rate (493 [915] vs 371 [349] per 100 000), and a higher mean (SD) county poverty rate (16.5% [5.4%] vs 14.8% [5.1%]) (Table). The mean (SD) county jail population rate for the entire cohort was 372 (358) per 100 000 population. The mean TJPR per 100 000 population for each of the nearly 2900 counties in the Vera Institute of Justice data from 2008 to 2018 was divided into quartiles. Figure 1A depicts the TJPR quartiles (1 being the lowest and 4 being the highest) across the country. During the 2008 to 2019 study period, 431 000 (11.6%) died of any cause and 5500 (0.2%) died of overdose (Table). Figure 1B and 1C depict quartiles of all-cause mortality and mortality from overdose, respectively, across the country.

**Figure 2.**
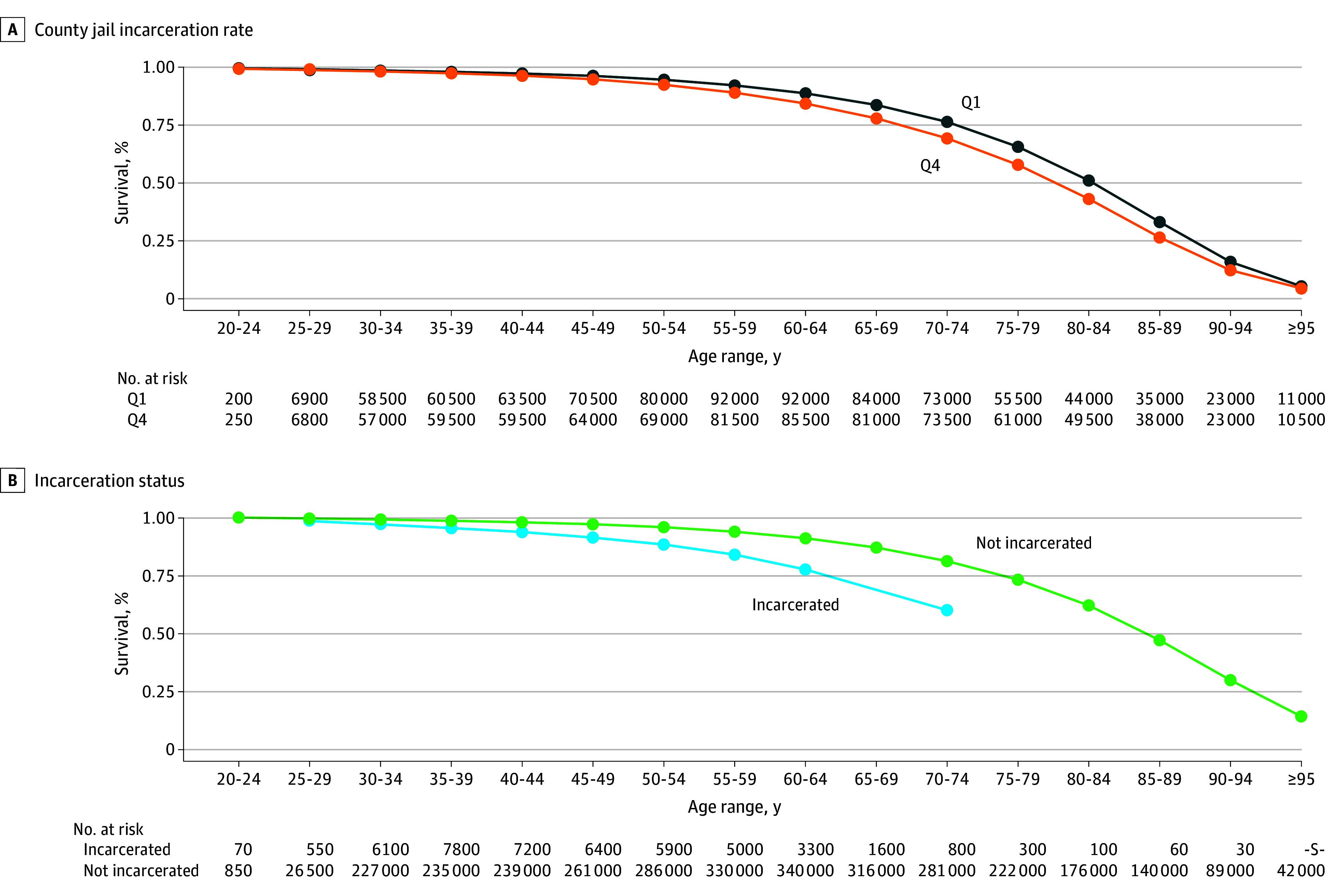
Cumulative Unadjusted Survival Rates at 5-Year Age Intervals for All-Cause Mortality Results approved by US Census Bureau Disclosure Review Board (approval number CBDRB-FY24-CES028-003). Q indicates quartile (Q1 is the lowest and Q4 the highest); S, suppressed.

In unadjusted analysis, cumulative survival rates at 5-year age intervals varied by TJPR quartile (quartile 1 vs quartile 4) and incarceration status (Figure 2). For the group aged 30 to 34 years, survival was 99.0% for nonincarcerated individuals and 96.9% for the incarcerated group (Figure 2). By 70 to 74 years of age, the difference was 20 percentage points lower in the incarcerated group.

**Figure 3.**
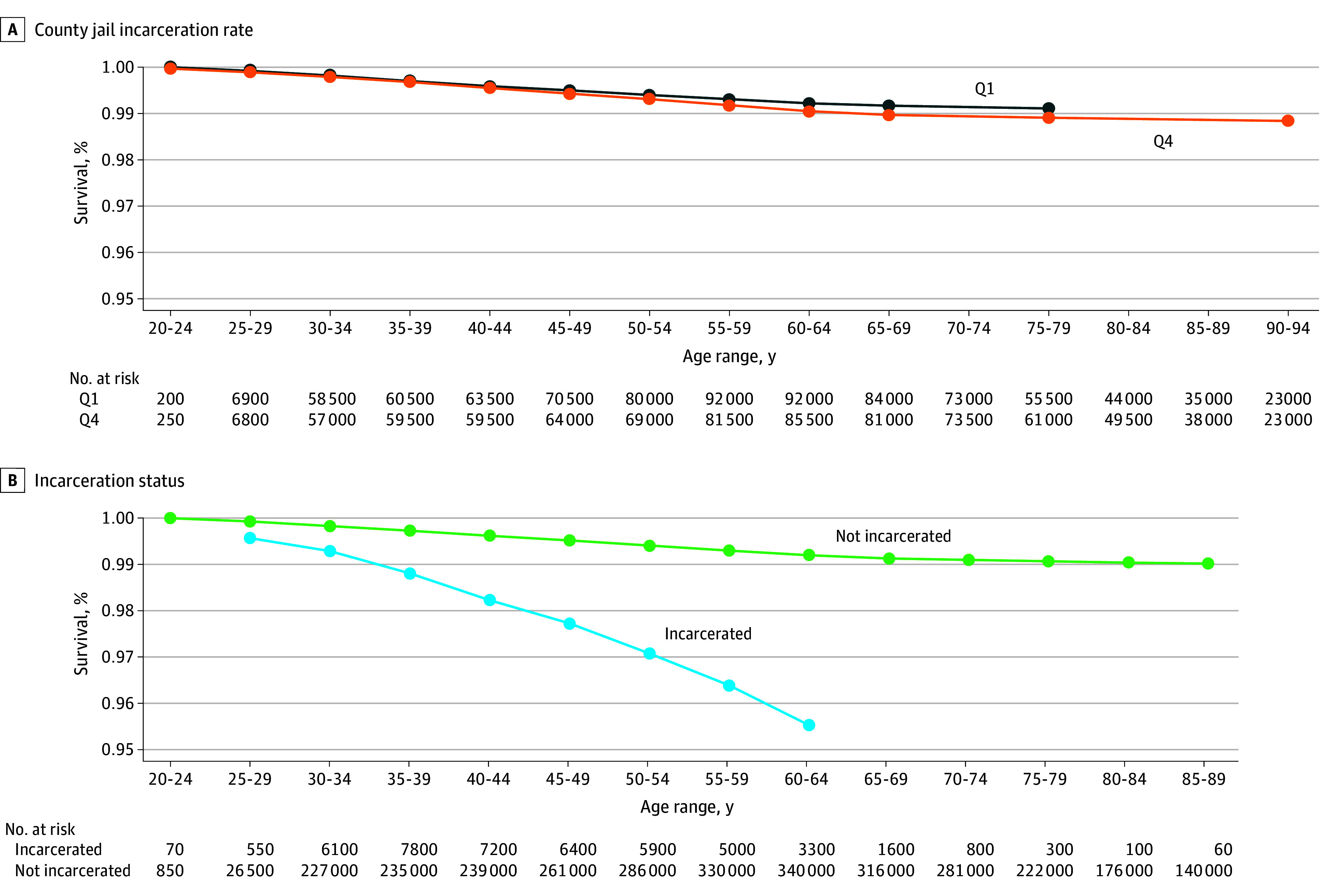
Cumulative Unadjusted Survival Rates at 5-Year Age Intervals for Overdose Mortality Results approved by US Census Bureau Disclosure Review Board (approval number CBDRB-FY24-CES028-003). Q indicates quartile (Q1 is the lowest and Q4 the highest).

We also analyzed overdose mortality by TJPR quartile and incarceration status at 5-year age intervals (Figure 3). While survival rates for quartiles 1 and 4 were similar, incarcerated individuals had lower survival at each age interval shown.

eTable 2 in Supplement 1 presents the results of weighted multivariable Cox proportional hazards survival analysis for all-cause mortality. Model 1 shows the hazard rate (HR) for the natural logarithm of county-level TJPR (ln[TJPR]) without any adjustments for other covariates. Model 2 adds adjustments for individual demographic and socioeconomic variables but does not adjust for county-level covariates. Model 3 controls for both individual- and county-level characteristics. Nested log likelihood tests and Wald χ^2^ tests on the models show that the goodness of fit for model 3 is better than for model 2, and both fit the data better than the univariable model 1.

**Figure 4.**
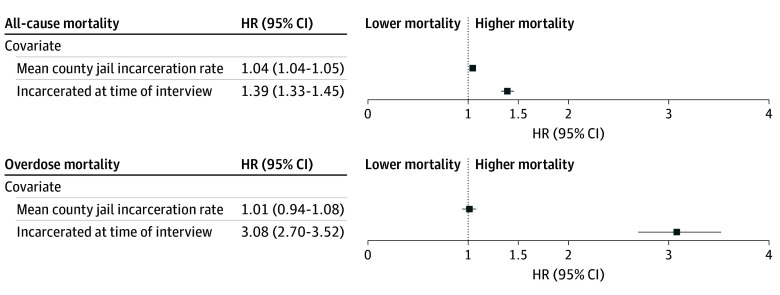
Weighted Multivariate Cox Proportional Hazards Survival Analysis Findings are adjusted for sex, age, race and ethnicity, marital status, educational attainment, employment, household income, county racial composition, county mean household size, county population per square mile, and county poverty rate. Results approved by US Census Bureau Disclosure Review Board (approval number CBDRB-FY24-CES028-003). HR indicates hazard rate.

In our fully adjusted model, all-cause mortality rates were lower for female relative to male individuals (HR, 0.64; 95% CI, 0.63-0.64). The association of aging with individual mortality was mostly explained by the other demographic and socioeconomic covariates. Relative to persons who were not incarcerated, mortality was higher for those who were incarcerated at the time of interview (incarcerated HR, 1.39; 95% CI, 1.33-1.45) (Figure 4). Of all the individual-level characteristics included in the model, the only characteristics associated with a higher hazard of all-cause mortality than incarcerated status were those related to being divorced, separated, or never married compared with being married (HR for divorced, 1.40 [95% CI, 1.38-1.41]; HR for separated, 1.44 [95% CI, 1.39-1.48]; HR for never married, 1.51 [95% CI, 1.48-1.53]). Higher mean TJPRs in a county were also associated with higher all-cause mortality rates. A county with a 10% higher mean jail population rate (eg, from a mean [SD] of 372 [358] to a hypothetical mean of 410) would have an increased expected all-cause mortality rate of about 0.45% (ln[TJPR] HR, 1.05 [95% CI, 1.04-1.05). Stated differently, this equates to 4.6 (95% CI, 3.8-5.5) additional people dying per 100 000 population for every 10% increase in incarceration rates.

eTable 3 in Supplement 1 presents the results of weighted multivariate Cox proportional hazards survival analysis on overdose mortality. The HR for those incarcerated in 2008 was 3.08 (95% CI, 2.70-3.52) (Figure 4), so that overdose mortality rates were about 3 times higher than for those who were not incarcerated at the time of the 2008 ACS interview. No other characteristic, individual- or area-level, was associated with a higher risk for overdose mortality than incarceration status. The county-level variables for 2008 to 2018 mean TJPR (Figure 4), population density, and mean household size were not associated with overdose mortality rates.

## Discussion

In this nationally representative cohort study, we examined associations between individual- and county-level characteristics and all-cause and overdose mortality in the US. Individual incarceration status was associated with a 39% higher risk of all-cause mortality and a 3-fold increase in overdose mortality compared with no incarceration. County-level incarceration rates posed additional all-cause mortality risk, even after controlling for individual incarceration and other county-level variables, but were not associated with overdose mortality.

Our results provide the most comprehensive US-based cohort study comparing mortality risks among those with and without incarceration history. While previous studies demonstrated increased mortality linked to individual- or area-level incarceration separately, ours is the first to examine these associations on both levels together on a national scale.^10,11,12^ Even after adjusting for area-level confounders, the association between county incarceration rates and all-cause mortality persisted.

County incarceration rate was linked to a higher hazard ratio of all-cause mortality than county racial composition or poverty rates, factors previously associated with mortality.^18,19,20,21,22^ However, no area-level factors, including county incarceration rate, were associated with an increased individual overdose mortality risk. A study by Norsati et al^23^ found county-level incarceration was associated with drug use–related mortality on the population level. There are several potential explanations for why county incarceration rate does not confer additional overdose mortality risk. Counties with higher jail incarceration rates may have higher treatment availability or mandatory treatment models, attenuating the impact of county incarceration rate on individual mortality risk. Our data suggest previous associations identified between county incarceration rates and mortality are primarily driven by the direct impact of incarceration on individual overdose mortality. Indirect harms of area-level incarceration, such as disruption of social networks or harm to family well-being, may contribute to the increased all-cause mortality our analysis revealed.

The link between incarceration and mortality is increasingly critical amid the overdose crisis and the need to address social and structural health determinants. Criminalization of addiction remains common, with nearly 50% of incarcerated people having substance use disorders.^24^ Recognizing incarceration as both a barrier to health care and an opportunity to reach individuals with limited access, policymakers are advancing reforms.^25,26^ In April 2023, the Centers for Medicare & Medicaid Services encouraged states to test Medicaid Section 1115 demonstration strategies for community reentry.^27^ To date, 11 states have approval to provide prerelease services to certain incarcerated, Medicaid-eligible individuals up to 90 days prior to release from incarceration.^28^ This policy marks the first time Medicaid has extended eligibility to incarcerated individuals and is expected to improve prerelease health care access, postrelease insurance coverage, and postrelease care coordination and potentially reduce mortality. A cohort study found that Medicaid expansion in Rhode Island, compared with nonexpansion in North Carolina, was associated with sustained reductions in all-cause mortality, primarily from overdose and homicide.^29^ These findings suggest Medicaid reforms may help reduce mortality among formerly incarcerated populations.

Studies within the last 10 years^30,31,32^ have highlighted the heightened risk of overdose mortality following incarceration, particularly within the first 2 weeks of release. Meta-analyses and international data underscore the effectiveness of medications for opioid use disorder (MOUD) in mitigating this risk. In Norway, where prison health care is integrated with the national system, MOUD have been associated with significant reductions in postrelease mortality.^33^ A systematic review^34^ found that individuals receiving MOUD had substantially lower all-cause and drug-related mortality within the first 4 weeks after release. Similarly, research in New York City jails^35^ reported an 80% reduction in overdose and all-cause mortality among individuals receiving methadone or buprenorphine. Despite this strong empirical support, MOUD implementation remains limited in US correctional settings. A recent study^36^ found that few US jails offer frontline treatments, highlighting a critical gap in care. Expanding and implementing existing, proven treatments—particularly MOUD—within US jails and prisons is essential to improving postrelease outcomes and reducing overdose-related deaths.

Much current policy and research on incarceration focuses on preventing overdose deaths, which our findings confirm are more common among individuals with incarceration histories. However, studies suggest noncommunicable diseases and suicide are also significant causes of death, particularly with longer follow-up.^37,38^ Our finding that county incarceration rates contribute to excess all-cause mortality highlights the urgency of improving health care during and after incarceration while supporting communities impacted by incarceration. Strengthening community-based primary care, shown to reduce population-level mortality, may help mitigate the impacts of high incarceration rates and reduce the mortality associated with individual- and county-level incarceration.^4,39^

### Strengths and Limitations

Strengths of our study include the large, nationally representative sample; the prospective 11-year follow-up period; and detailed covariates. However, notable limitations exist. Dataset limitations include potential misclassification of mortality, inability to control for underlying medical and psychiatric comorbidities, the ascertainment of time-varying measures including the availability of the primary exposure of interest only at baseline, and lack of information on incarceration duration. The broad definition of correctional facilities in the ACS, which includes jails, prisons, halfway houses, restitution centers, and work release centers, may mask differences in health risks across these settings, though all settings likely represent recent incarceration in a carceral facility.

Our analysis was limited to all-cause and overdose mortality and did not explore other cause-specific mortality. We did not stratify results by race and ethnicity, limiting inferences on racial and ethnic disparities.^40^ This should be an area of future research. While our models account for many potential confounders, our analyses have the same weakness as all secondary data analyses where unmeasured confounding remains possible. These results should be interpreted as associations rather than causal effects. Future research using quasi-experimental designs may better estimate causal effects.

## Conclusions

In this cohort study of 3.26 million individuals in the US, incarceration at both the individual and area levels was associated with increased risk of all-cause mortality. These findings highlight the critical need for changes in policy and practice to promote health care access among individuals and communities impacted by incarceration. Area-level incarceration rates should be further examined as contributors to premature mortality. Our study underscores the urgent need to develop interventions to reduce mortality and health inequities associated with policies of mass incarceration.

## Notes

Supplement 1. **eTable 1.** Definitions of Variables **eTable 2.** Hazard Ratios (95% CIs) From Cox Regression Models for All-Cause Mortality (N = 3 255 000) **eTable 3.** Hazard Ratios From Cox Regression Models for Overdose Mortality (n = 3 323 000)

Supplement 2.
Data Sharing Statement

## References

[1] Sawyer W, Wagner P. Mass incarceration: the whole pie 2024. Prison Policy Initiative. Accessed March 14, 2024. https://www.prisonpolicy.org/reports/pie2024.html

[2] Bureau of Justice Statistics. Correctional populations in the United States, 2022—statistical tables. Report No.: NCJ 308699. Accessed May 1, 2024. https://bjs.ojp.gov/library/publications/correctional-populations-united-states-2022-statistical-tables

[3] Western B. Punishment and Inequality in America. Russell Sage Press; 2006.

[4] Wildeman C, Wang EA. Mass incarceration, public health, and widening inequality in the USA. Lancet. 2017;389(10077):1464-1474. doi:10.1016/S0140-6736(17)30259-3 28402828

[5] Mauer M. Incarceration Rates in an International Perspective. Oxford University Press; 2017.

[6] Roberts DE. The social and moral cost of mass incarceration in African American communities. Stanford Law Rev. 2004;56(5):1271-1305.

[7] Nowotny KM, Kuptsevych-Timmer A. Health and justice: framing incarceration as a social determinant of health for Black men in the United States. Sociol Compass. 2018;12(3):e12566. doi:10.1111/soc4.12566

[8] Sundaresh R, Yi Y, Harvey TD, . Exposure to family member incarceration and adult well-being in the United States. JAMA Netw Open. 2021;4(5):e2111821. doi:10.1001/jamanetworkopen.2021.11821 34047791 PMC8164096

[9] Weidner RR, Schultz J. Examining the relationship between US incarceration rates and population health at the county level. SSM Popul Health. 2019;9:100466. doi:10.1016/j.ssmph.2019.100466 31485477 PMC6715952

[10] Binswanger IA, Stern MF, Deyo RA, . Release from prison–a high risk of death for former inmates. N Engl J Med. 2007;356(2):157-165. doi:10.1056/NEJMsa064115 17215533 PMC2836121

[11] Ranapurwala SI, Shanahan ME, Alexandridis AA, . Opioid overdose mortality among former North Carolina inmates: 2000-2015. Am J Public Health. 2018;108(9):1207-1213. doi:10.2105/AJPH.2018.304514 30024795 PMC6085027

[12] Kajeepeta S, Mauro PM, Keyes KM, El-Sayed AM, Rutherford CG, Prins SJ. Association between county jail incarceration and cause-specific county mortality in the USA, 1987-2017: a retrospective, longitudinal study. Lancet Public Health. 2021;6(4):e240-e248. doi:10.1016/S2468-2667(20)30283-8 33636104 PMC8054445

[13] Brinkley-Rubinstein L. Incarceration as a catalyst for worsening health. Health Justice. 2013;1(1):3. doi:10.1186/2194-7899-1-3

[14] US Census Bureau. Mortality Disparities in American Communities (MDAC). Updated September 20, 2022. Accessed October 3, 2022. https://www.census.gov/mdac

[15] Kang-Brown J. Incarceration trends: data and methods for historical jail populations in U.S. counties, 1970-2014. Vera Institute of Justice. December 2015. Accessed November 1, 2021. https://www.prisonlegalnews.org/media/publications/Incarceration%20Trends%20-%20Historical%20Jail%20Populations%201970-2014%2C%20Vera%20Institute%20of%20Justice%2C%202015.pdf

[16] US Census Bureau. American Community Survey 5-year data. December 12, 2024. Accessed April 29, 2025. https://www.census.gov/data/developers/data-sets/acs-5year.html

[17] Ahmad FBCJ, Rossen LM, Sutton P. Provisional drug overdose death counts. National Center for Health Statistics. 2024. Accessed November 1, 2024. https://www.cdc.gov/nchs/nvss/vsrr/drug-overdose-data.htm

[18] Frankenfeld CL, Hakes JK, Leslie TF. All-cause mortality and residential racial and ethnic segregation and composition as experienced differently by individual-level race, ethnicity, and gender: mortality disparities in American communities data. Ann Epidemiol. 2022;65:38-45. doi:10.1016/j.annepidem.2021.10.008 34757014

[19] McLaughlin DK, Stokes CS. Income inequality and mortality in US counties: does minority racial concentration matter? Am J Public Health. 2002;92(1):99-104. doi:10.2105/AJPH.92.1.99 11772770 PMC1447397

[20] Gordon SH, Sommers BD. Recessions, poverty, and mortality in the United States: 1993-2012. Am J Health Econ. 2016;2(4):489-510. doi:10.1162/AJHE_a_00060

[21] Cosby AG, McDoom-Echebiri MM, James W, Khandekar H, Brown W, Hanna HL. Growth and persistence of place-based mortality in the United States: the rural mortality penalty. Am J Public Health. 2019;109(1):155-162. doi:10.2105/AJPH.2018.304787 30496008 PMC6301407

[22] Monnat SM. Factors associated with county-level differences in US drug-related mortality rates. Am J Prev Med. 2018;54(5):611-619. doi:10.1016/j.amepre.2018.01.040 29598858 PMC6080628

[23] Nosrati E, Kang-Brown J, Ash M, McKee M, Marmot M, King LP. Economic decline, incarceration, and mortality from drug use disorders in the USA between 1983 and 2014: an observational analysis. Lancet Public Health. 2019;4(7):e326-e333. doi:10.1016/S2468-2667(19)30104-5 31279417

[24] Bronson J, Stroop J. Drug use, dependence, and abuse among state prisoners and jail inmates, 2007-2009. Bureau of Justice Statistics. 2017. Accessed October 3, 2024. https://bjs.ojp.gov/content/pub/pdf/dudaspji0709.pdf

[25] Zhao J, Star J, Han X, . Incarceration History and Access to and Receipt of Health Care in the US. JAMA Health Forum. 2024;5(2):e235318. doi:10.1001/jamahealthforum.2023.5318 38393721 PMC10891474

[26] Kendig NE, Butkus R, Mathew S, Hilden D; Health and Public Policy Committee of the American College of Physicians. Health care during incarceration: a policy position paper from the American College of Physicians. Ann Intern Med. 2022;175(12):1742-1745. doi:10.7326/M22-2370 36410006

[27] Tsai D. RE: Opportunities to test transition-related strategies to support community reentry and improve care transitions for individuals who are incarcerated. April 17, 2023. Accessed November 3, 2024. https://www.medicaid.gov/federal-policy-guidance/downloads/smd23003.pdf

[28] Hinton E, Pillai A, Amaya D. Section 1115 waiver watch: Medicaid pre-release services for people who are incarcerated. Kaiser Family Foundation. August 19, 2024. Accessed November 19, 2024. https://www.kff.org/medicaid/issue-brief/section-1115-waiver-watch-medicaid-pre-release-services-for-people-who-are-incarcerated/

[29] Perera PS, Miller VE, Fitch KV, . Medicaid expansion and mortality among persons who were formerly incarcerated. JAMA Netw Open. 2024;7(9):e2429454. doi:10.1001/jamanetworkopen.2024.29454 39287949 PMC11409152

[30] Malta M, Varatharajan T, Russell C, Pang M, Bonato S, Fischer B. Opioid-related treatment, interventions, and outcomes among incarcerated persons: a systematic review. PLoS Med. 2019;16(12):e1003002. doi:10.1371/journal.pmed.1003002 31891578 PMC6938347

[31] Marsden J, Stillwell G, Jones H, . Does exposure to opioid substitution treatment in prison reduce the risk of death after release? a national prospective observational study in England. Addiction. 2017;112(8):1408-1418. doi:10.1111/add.13779 28160345

[32] Bird SM, Fischbacher CM, Graham L, Fraser A. Impact of opioid substitution therapy for Scotland’s prisoners on drug-related deaths soon after prisoner release. Addiction. 2015;110(10):1617-1624. doi:10.1111/add.12969 25940815 PMC4744745

[33] Bukten A, Stavseth MR. Estimated effects of opioid agonist treatment in prison on all-cause mortality and overdose mortality in people released from prison in Norway: a prospective analysis of data from the Norwegian Prison Release Study (nPRIS). Lancet Public Health. 2024;9(7):e421-e431. doi:10.1016/S2468-2667(24)00098-7 38942554

[34] Santo T Jr, Clark B, Hickman M, . Association of opioid agonist treatment with all-cause mortality and specific causes of death among people with opioid dependence: a systematic review and meta-analysis. JAMA Psychiatry. 2021;78(9):979-993. doi:10.1001/jamapsychiatry.2021.0976 34076676 PMC8173472

[35] Lim S, Cherian T, Katyal M, . Association between jail-based methadone or buprenorphine treatment for opioid use disorder and overdose mortality after release from New York City jails 2011-17. Addiction. 2023;118(3):459-467. doi:10.1111/add.16071 36305669 PMC9898114

[36] Flanagan Balawajder E, Ducharme L, Taylor BG, . Factors associated with the availability of medications for opioid use disorder in US jails. JAMA Netw Open. 2024;7(9):e2434704. doi:10.1001/jamanetworkopen.2024.34704 39316401 PMC11423166

[37] Mundt AP, Cifuentes-Gramajo PA, Baranyi G, Fazel S. Worldwide incidence of suicides in prison: a systematic review with meta-regression analyses. Lancet Psychiatry. 2024;11(7):536-544. doi:10.1016/S2215-0366(24)00134-2 38823401

[38] Borschmann R, Mortality After Release From Incarceration Consortium (MORIAC) collaborators; Kinner SA. Rates and causes of death after release from incarceration among 1,471,526 people in eight high-income and middle-income countries: an individual participant data meta-analysis. Lancet. 2024;403(10438):1779-1788. doi:10.1016/S0140-6736(24)00344-1 38614112

[39] Cloud DH, Garcia-Grossman IR, Armstrong A, Williams B. Public health and prisons: priorities in the age of mass incarceration. Annu Rev Public Health. 2023;44:407-428. doi:10.1146/annurev-publhealth-071521-034016 36542770 PMC10128126

[40] Bigham Z, Boms O, Guardado R, Bunn DA, Glenn JE, Wurcel AG. Increased mortality of Black incarcerated and hospitalized people: a single state cohort analysis. J Racial Ethn Health Disparities. 2024;11(5):2973-2980. doi:10.1007/s40615-023-01755-7 37672188

