# Supplementary material for: Individual- and Area-Level Incarceration and Mortality
Source: JAMA Netw Open. 2025 Jun 3;8(6):e2513537. doi: 10.1001/jamanetworkopen.2025.13537 (PMC12134954; doi:10.1001/jamanetworkopen.2025.13537)
Supplement: Supplement 1. — eTable 1. Definitions of Variables eTable 2. Hazard Ratios (95% CIs) From Cox Regression Models for All-Cause Mortality (N = 3 255 000) eTable 3. Hazard Ratios From Cox Regression Models for Overdose Mortality (n = 3 323 000) [file jamanetwopen-e2513537-s001.pdf]

Supplementary Online Content

Khatri UG, Hakes JK, Buckler D, Zebrowski A, Winkelman T. Individual- and area-level incarceration and mortality in the US. *JAMA Netw Open.* 2025;8(6):e2513537. doi:10.1001/jamanetworkopen.2025.13537

eTable 1. Definitions of Variables

eTable 2. Hazard Ratios (95% CIs) from Cox Regression Models for All-Cause Mortality (N = 3 255 000)

eTable 3. Hazard Ratios From Cox Regression Models for Overdose Mortality (N = 3 323 000)

This supplementary material has been provided by the authors to give readers additional information about their work.

© 2025 Khatri UG et al. *JAMA Network Open*.

eTable 1. Definitions of Variables

**<u>Individual Characteristics</u>**	
**Sex**<br><br>Female	Respondent selected female in ACS*
Male	Respondent selected male in ACS
**Age**	
18-25	Age (collected as a continuous variable) in this range in ACS
26-35	Age (collected as a continuous variable) in this range in ACS
36-45	Age (collected as a continuous variable) in this range in ACS
46-65	Age (collected as a continuous variable) in this range in ACS
66-85	Age (collected as a continuous variable) in this range in ACS
86 and up	Age (collected as a continuous variable) in this range in ACS
**Self-Identified Race/Ethnicity**	
Hispanic	Respondent selected Cuban, Mexican, Mexican Am, Chicano, Puerto Rican, South or Central American, or another Hispanic, Latino, or Spanish origin, regardless of race in ACS
Non-Hispanic Black	Respondent selected Black or African American as their race and explicitly stated they are not of Hispanic or Latino origin in ACS
Non-Hispanic White	Respondent selected White as their race and explicitly stated they are not of Hispanic or Latino origin in ACS
Non-Hispanic Asian	Respondent selected Asian as their race and explicitly stated they are not of Hispanic or Latino origin in ACS

© 2025 Khatri UG et al. *JAMA Network Open*.

Non-Hispanic American Indian or Alaskan Native	Respondent selected American-Indian or Alaskan Native as their race and explicitly stated they are not of Hispanic or Latino origin in ACS
Other	Respondent selected race category other than those above and explicity stated they are not of Hispanic or Latino origin in ACS
**Marital Status**	Categorizes individuals based on their current legal marital status in ACS
Married	Respondent selected now married in ACS
Widowed	Respondent selected widowed and not remarried in ACS
Divorced	Respondent selected divorced and not currently married in ACS
Separated	Respondent selected separated in ACS
Never Married	Respondent selected never married in ACS
**Education**	Categorizes individuals based on the highest level of education completed.
Less than high school	Aggregated category of respondent selection to include no schooling completed, nursery, kidergarten or first grade, Grades 1-4, Grades 5-8, Grades 9-12 (No Dipoloma) in ACS
High school diploma	Respondent selected High School Graduate and equivalency (GED) in ACS
Some college	Respondent selected Attended some college, no degree in ACS
Degree	Aggregated catetgory for respondent selection to include associate degree, Bachelor's degree, Master's degree, professional degree in ACS
**Employment**	Categorizes individuals based on their labor force participation in ACS

© 2025 Khatri UG et al. *JAMA Network Open*.

Unemployed	Respondent selected they were not working but actively looking for work in the past four weeks and available to start a job in ACS
Employed	Respondent selected they worked for pay or profit during the reference week or those who had a job inidcated they were temporarily absent
**Incarcerated at interview**	Identified through Group Quarters Variable in ACS coded as "Correctional Facilities for Adults"
**Household Poverty**	Household poverty status is determined based on poverty thresholds set annually by the U.S. Census Bureau. The ACS assigns poverty status to each household based on the total income of the household relative to these thresholds.
Less than 100%	Household income is below the federal poverty threshold (considered in poverty).
100-199%	Household income between 100-199% above poverty threshold
200-399%	Household income between 200-399% above poverty threshold
400-899%	Household income between 400-899% above poverty threshold
900% and up	Household income 900% above poverty threshold or higher
**Deceased**	Death from any cause identified in the National Death Index
**Overdose death**	Death in the National Death Index from drug overdose mortality, identified using Centers for Disease Control and Prevention definitions and ICD–10 codes X40–X44, X60–X64, X85, and Y10–Y14

© 2025 Khatri UG et al. *JAMA Network Open*.

**County Characteristics**	
Percent of Population that is Black	Proportion of residents at a county or county-equivalent (using FIPS code) reporting their race/ethnicity as Black per ACS survey
Average total jail population rate (per 100,000, 2008-2018)	Average number of people held in jail per 100,000 residents in a given county over a specified period
Average Household Size	Mean Total population in households divided by total number of households at county or county-equivalent (using FIPS code)
Population per square mile	Population density of a FIPS code. It is calculated by dividing the total population of the area by its total land area in square miles.
County Poverty Rate	Percentage of people in a county whose income falls below 100% of the federal poverty threshold, as defined by the U.S. Census Bureau.

eTable 2. Hazard Ratios (95% CIs) from Cox Regression Models for All-Cause Mortality (N = 3 255 000)

	Model 1<br>*TJPR*	*Model 2*<br>*TJPR + Individual Characteristics*	*Model 3*<br>*TJPR + Individual Characteristics + County Characteristics*
ln(Average Total Jail Population Rate, 2008-2018)	1.157***<br>(1.150, 1.165)	1.087***<br>(1.080, 1.094)	1.045***<br>(1.037, 1.053)
Individual Characteristics			
Incarcerated at time of interview		1.402***<br>(1.343, 1.463)	1.391***<br>(1.333, 1.452)
Female<br>Reference group: Male		0.635***<br>(0.629, 0.640)	0.635***<br>(0.630, 0.640)

© 2025 Khatri UG et al. *JAMA Network Open*.

Age<br>Reference group: 18-25			
26-35		1.000<br>(0.932, 1.073)	1.003<br>(0.935, 1.076)
36-45		0.978<br>(0.895, 1.070)	0.982<br>(0.898, 1.074)
46-65		1.064<br>(0.967, 1.171)	1.068<br>(0.970, 1.175)
66-85		1.062<br>(0.964, 1.171)	1.066<br>(0.967, 1.175)
86 and up		1.016<br>(0.921, 1.122)	1.020<br>(0.924, 1.126)
Race/ethnicity<br>Reference group:<br>Non-Hispanic White			
Hispanic		0.573***<br>(0.562, 0.584)	0.577***<br>(0.567, 0.588)
Non-Hispanic Black		0.928***<br>(0.915, 0.941)	0.922***<br>(0.908, 0.936)
Non-Hispanic American Indian or Alaskan Native		1.124***<br>(1.067, 1.184)	1.103***<br>(1.047, 1.162)
Non-Hispanic Asian		0.729***<br>(0.710, 0.748)	0.747***<br>(0.727, 0.767)
Other		1.031<br>(0.987, 1.078)	1.032<br>(0.987, 1.078)
Marital Status<br>Reference group: Married			
Widowed		1.229***<br>(1.215, 1.242)	1.228***<br>(1.215, 1.241)
Divorced		1.395***<br>(1.378, 1.412)	1.396***<br>(1.379, 1.413)
Separated		1.430***	1.438***

© 2025 Khatri UG et al. *JAMA Network Open*.

		(1.386, 1.476)	(1.393, 1.484)
Never Married		1.496***<br>(1.473, 1.519)	1.505***<br>(1.483, 1.529)
Education<br>Reference group:<br>High school diploma			
Less than high school		1.101***<br>(1.090, 1.113)	1.100***<br>(1.088, 1.112)
Some College		0.897***<br>(0.888, 0.906)	0.896***<br>(0.887, 0.905)
Degree		0.692***<br>(0.684, 0.700)	0.693***<br>(0.685, 0.701)
Household Income (as Percent of Poverty Line) Reference Group 100-199%			
Less than 100%		1.113***<br>(1.097, 1.129)	1.114***<br>(1.098, 1.130)
200-399%		0.891***<br>(0.882, 0.901)	0.893***<br>(0.884, 0.903)
400-899%		0.753***<br>(0.745, 0.762)	0.758***<br>(0.749, 0.767)
900% and up		0.612***<br>(0.601, 0.623)	0.618***<br>(0.607, 0.629)
County Characteristics			
Percent of Population that is Black (%)			1.0008***<br>(1.0004, 1.0011)
Average Household Size (persons)			0.983*<br>(0.966, 0.999)
Population square mile (100,000 persons/sq. mile)			0.644***<br>(0.619, 0.670)

© 2025 Khatri UG et al. *JAMA Network Open*.

County Poverty Rate (%)			1.0056***<br>(1.0048, 1.0066)
Observations,<br>*Deaths,*<br>*Wald chi2(with 1, 24, 28 d.f.)*	3,255,000<br>431,000<br>1850	3,255,000<br>431,000<br>38720	3,255,000<br>431,000<br>39220

Notes: *- p in (0.01,0.05), ** - p in (0.001, 0.01), *** - p < 0.001.

Results approved by U.S. Census Bureau Disclosure Review Board (Project 7530062: CBDRB-FY24-CES028-003).

© 2025 Khatri UG et al. *JAMA Network Open*.

eTable 3. Hazard Ratios From Cox Regression Models for Overdose Mortality (N = 3 323 000)

	Model 1<br>*TJPR*	*Model 2*<br>*TJPR + Individual Characteristics*	*Model 3*<br>*TJPR + Individual Characteristics + County Characteristics*
ln(Average Total Jail Population Rate, 2008-2018)	1.152***<br>(1.084, 1.224)	0.989<br>(0.933, 1.049)	1.007<br>(0.942, 1.076)
Individual Characteristics			
Incarcerated at time of interview		3.059***<br>(2.680, 3.491)	3.080***<br>(2.699, 3.516)
Female<br>Reference group: Male		0.612***<br>(0.571, 0.656)	0.611***<br>(0.570, 0.654)
Age<br>Reference group: 18-25			
26-35		0.773**<br>(0.663, 0.903)	0.772**<br>(0.662, 0.902)
36-45		0.549***<br>(0.442, 0.682)	0.547***<br>(0.440, 0.680)
46-65		0.449***<br>(0.347, 0.581)	0.448***<br>(0.346, 0.580)
66-85		0.279***<br>(0.181, 0.429)	0.278***<br>(0.181, 0.428)
86 and up		0.215***<br>(0.091, 0.510)	0.214***<br>(0.090, 0.508)
Race/ethnicity<br>Reference group:<br>Non-Hispanic White			
Hispanic		0.289***<br>(0.252, 0.333)	0.292***<br>(0.252, 0.339)
Non-Hispanic Black		0.429***<br>(0.381, 0.482)	0.408***<br>(0.359, 0.464)

© 2025 Khatri UG et al. *JAMA Network Open*.

Non-Hispanic American Indian or Alaskan Native		0.930 (0.664, 1.303)	0.983 (0.703, 1.373)
Non-Hispanic Asian		0.223*** (0.158, 0.316)	0.221*** (0.156, 0.314)
Other		0.946 (0.750, 1.195)	0.946 (0.749, 1.194)
Marital Status *Reference group: Married*			
Widowed		2.336*** (1.859, 2.935)	2.329*** (1.854, 2.927)
Divorced		2.617*** (2.379, 2.880)	2.612*** (2.374, 2.875)
Separated		2.699*** (2.276, 3.201)	2.686*** (2.265, 3.186)
Never Married		2.348*** (2.134, 2.583)	2.332*** (2.119, 2.566)
Education Reference group: High school diploma			
Less than high school		1.153** (1.048, 1.270)	1.155** (1.049, 1.271)
Some College		0.822*** (0.759, 0.891)	0.822*** (0.759, 0.891)
Degree		0.381*** (0.337, 0.430)	0.377*** (0.334, 0.426)
Household Income (as Percent of Poverty Line) Reference Group 100-199%			
Less than 100%		1.637*** (1.474, 1.817)	1.648*** (1.484, 1.830)
200-399%		0.773*** (0.698, 0.857)	0.766*** (0.692, 0.849)

© 2025 Khatri UG et al. *JAMA Network Open*.

400-899%		0.647***<br>(0.579, 0.723)	0.634***<br>(0.567, 0.709)
900% and up		0.597***<br>(0.495, 0.718)	0.579***<br>(0.481, 0.698)
County Characteristics			
Percent of Population that is Black (%)			1.005**<br>(1.002, 1.008)
Average Household Size (persons)			0.999<br>(0.860, 1.160)
Population square mile (100,000 persons/sq. mile)			0.980<br>(0.684, 1.350)
County Poverty Rate (%)			0.988**<br>(0.980, 0.995)
Observations,<br>*Deaths,*<br>*Wald chi2(with 1, 27, 31 d.f.)*	3,255,000<br>5,500<br>21.0	3,255,000<br>5,500<br>3479	3,255,000<br>5,500<br>3503

Notes: *- p in (0.01,0.05), ** - p in (0.001, 0.01), *** - p < 0.001.

Results in Figure approved by U.S. Census Bureau Disclosure Review Board (Project 7530062: CBDRB-FY24-CES028-003).

© 2025 Khatri UG et al. *JAMA Network Open*.
