# Supplementary material for: Individual- and Area-Level Incarceration and Mortality
Source: JAMA Netw Open. 2025 Jun 3;8(6):e2513537. doi: 10.1001/jamanetworkopen.2025.13537 (PMC12134954; doi:10.1001/jamanetworkopen.2025.13537)
Supplement: Supplement 2. — Data Sharing Statement [file jamanetwopen-e2513537-s002.pdf]

Data Sharing Statement

Khatri. Individual- and Area-Level Incarceration and Mortality in the US. *JAMA Netw Open*. Published June 03, 2025. doi:10.1001/jamanetworkopen.2025.13537

Data
Data available: No

Additional Information
Explanation for why data not available: Data sharing is limited due to the source from the MDAC project. The Census Bureau limits the use of confidential data and has reviewed these results for disclosure avoidance protection.
